# Episode clustering in phylogenetic networks

**DOI:** 10.1093/bioinformatics/btag445

**Published:** 2026-08-10

**Authors:** Paweł Górecki, Agnieszka Mykowiecka, Jarosław Paszek

**Affiliations:** Faculty of Mathematics, Informatics, and Mechanics, University of Warsaw, Warsaw 02-097, Poland; Faculty of Mathematics, Informatics, and Mechanics, University of Warsaw, Warsaw 02-097, Poland; Faculty of Mathematics, Informatics, and Mechanics, University of Warsaw, Warsaw 02-097, Poland

## Abstract

**Motivation:**

The classical duplication episode clustering (EC) model introduced by Guigó *et al.* in the 1990s provides a foundational approach for inferring genomic duplication events crucial to understanding genome evolution. This model clusters single gene duplications from a collection of gene trees at locations in the species tree to minimize the total number of such locations, called duplication episodes. However, it does not capture reticulate evolutionary histories.

**Results:**

Here, we introduce NetEC, a novel extension of this problem to phylogenetic networks. To solve NetEC, we first develop a polynomial-time dynamic programming (DP) algorithm for testing whether a given set of network nodes can serve as episode locations. We then propose a main inference algorithm that utilizes this DP component to optimize the episode count; while the feasibility test runs in polynomial time, the full optimization has exponential worst-case complexity, and an optional heuristic mode is provided for larger instances. We also propose an extended episode analysis procedure that identifies additional genomic duplication candidates below reticulation nodes, complementing the main algorithm by resolving potential upward clustering of duplications induced by reticulation. We evaluate our method on simulated data and on an empirical Pandanales dataset comprising over 29 000 gene trees, demonstrating exact and accurate inference of genomic duplication events even in the presence of multiple reticulations.

**Availability and implementation:**

All experiments were conducted using the NetEC tool (https://github.com/ppgorecki/netec), with all input data, scripts, and parameter settings for reproduction available in the same repository.

## 1 Introduction

Phylogenetic networks have emerged as a robust framework for representing complex evolutionary relationships (Huson *et al.* 2010) that traditional tree-based models cannot adequately capture. Unlike phylogenetic trees, networks accommodate reticulate events such as hybridization, horizontal gene transfer, and recombination, creating multiple pathways of inheritance.

Whole-genome duplications (WGDs) represent particularly significant evolutionary events that have shaped eukaryotic genomes (Ohno 1970, Kuzmin *et al.* 2020). In phylogenetic networks, WGDs introduce additional complexity: hybridization following independent WGDs in parental lineages generates intricate patterns of gene family evolution, as exemplified by polyploid plant and fungal lineages (Wolfe and Shields 1997, Salman-Minkov *et al.* 2016). Reliable inference of WGDs in such reticulate histories has direct applications in crop breeding, fungal genome analysis, and studies of vertebrate paleopolyploidy.

The concept of duplication episode clustering, developed initially for tree-based phylogenies (Guigó *et al.* 1996), aims to identify genomic locations where multiple gene duplications co-occurred, suggesting large-scale duplication events like WGDs. Informally, an *episode* is a node in the species phylogeny to which one or more gene duplications are assigned; episode clustering seeks the smallest set of such nodes explaining all observed duplications across a collection of gene trees. While episode clustering for trees has been extensively studied with efficient polynomial-time algorithms (Burleigh *et al.* 2008, Luo *et al.* 2011, Paszek and Górecki 2018a, 2018b), its extension to phylogenetic networks remains largely unexplored. A complementary inference problem, solved in polynomial time in (Iersel *et al.* 2020) under *weakly-displays*, asks for a network minimizing episodes or reticulations. We instead take the network as input and route each lineage through a single reticulation copy.

Extending episode clustering to networks introduces several theoretical and computational challenges. First, the presence of reticulation nodes means that gene duplications can be assigned to episodes along multiple alternative evolutionary histories, creating an exponential space of possible episode configurations. Second, the biological interpretation of episodes in networks requires careful consideration: should episodes be defined on the network structure itself, or on the individual tree-like scenarios (display trees) embedded within the network? Third, the computational complexity of network reconciliation suggests that episode clustering in networks may require fundamentally different algorithmic strategies than those used in tree-based approaches.

In our recent work (Górecki *et al.* 2024), we introduced a polynomial-time dynamic programming algorithm using three-valued logic for episode feasibility testing in the tree-based setting. Here we extend this approach to phylogenetic networks, where multiple alternative evolutionary histories through reticulation nodes complicate episode assignment.

In this work, we formalize NetEC (Network Episode Clustering), the problem of identifying duplication episodes in phylogenetic networks, and introduce the first algorithmic solution for it in the presence of reticulate evolution. Building on (Górecki *et al.* 2024), we extend the three-valued logic dynamic programming approach to handle multiple evolutionary scenarios in networks. While the previous approach resolves uncertainty about gene-species leaf assignments, here we show how to resolve uncertainty about which evolutionary paths through reticulation nodes are compatible with observed duplication patterns. Our approach leverages unfolded networks, a transformation expanding a network into a tree-like structure representing all scenario choices, and formulates the problem using functions that determine reticulation path choices.

The key contributions of this work are: (1) a formal definition of episode clustering for phylogenetic networks based on scenario-based gene-network reconciliation; (2) a polynomial-time algorithm for testing episode feasibility in networks via dynamic programming; (3) an exact algorithm for NetEC with practical heuristics for identifying optimal solutions; and (4) experimental validation demonstrating the method’s effectiveness in detecting WGDs in simulated and real biological networks with complex reticulate histories.

## 2 Basic definitions

A (phylogenetic) *network on a set of taxa* Θ is a directed acyclic graph N=(V(N),E(N)) such that (1) there is a unique node, called a *root*, such that there is a directed path from the root to any node in *N* and (2) leaves of *N* (i.e. nodes of indegree 1 and outdegree 0) are bijectively labelled by the elements from Θ. The leaf labelling is a function Λ:L(N)→Θ, where L(N) is the set of all leaves in *N*. A node of *N* is a reticulation if it has an indegree of 2. Nodes that are not leaves are *internal*; internal nodes that are not reticulations are called *tree nodes*. By R(N) we denote the set of all reticulations in *N*. If (s,t)∈E(N) then *s* is called a *parent* of *t* and *t* is called a *child* of *s*. A network is binary if its leaves, root, and the remaining nodes have degrees 1, 2 and 3, respectively. *N* is *semi-binary* if, in addition, it may contain semi-binary nodes with indegree at most 1 and outdegree 1, including the case where the root has exactly one child. A semi-binary node *v* of indegree 1 can be contracted by: (1) removing *v* and the edges incident with *v*, and (2) inserting a new edge connecting the parent of *v* with the child of *v*. If *v* has indegree 0, then after removing *v*, the child of *v* becomes the new root. We say that an edge (s,t)∈E(N) is a *reticulation edge* if t∈R(N). If a node *s* has exactly one child, then the child is denoted by $s^{\prime }$ and if *s* has exactly two children, then the children are denoted by $s^{\prime }$ and $s^{\prime \prime }$. In the latter case, we say that $s^{\prime }$ is a sibling of $s^{\prime \prime }$ and vice versa. If there is a directed path from *s* to *t*, then we say that *t* is *visible* from *s*, denoted as s≽t. A network on Θ is *tree-child* if every non-leaf node has a non-reticulation child. A *gene tree over a set of taxa* Θ is defined similarly to the network but with two differences: it has no reticulation nodes, and the leaf labelling Λ is not required to be a bijection.

### 2.1 Unfolded network

The unfolded network is a specific multi-labelled tree (MUL-tree, (Huber and Moulton 2006)) obtained by unfolding reticulation nodes. Here, we briefly recall the unfolding construction from (Wawerka *et al.* 2022).

**Figure 1:**
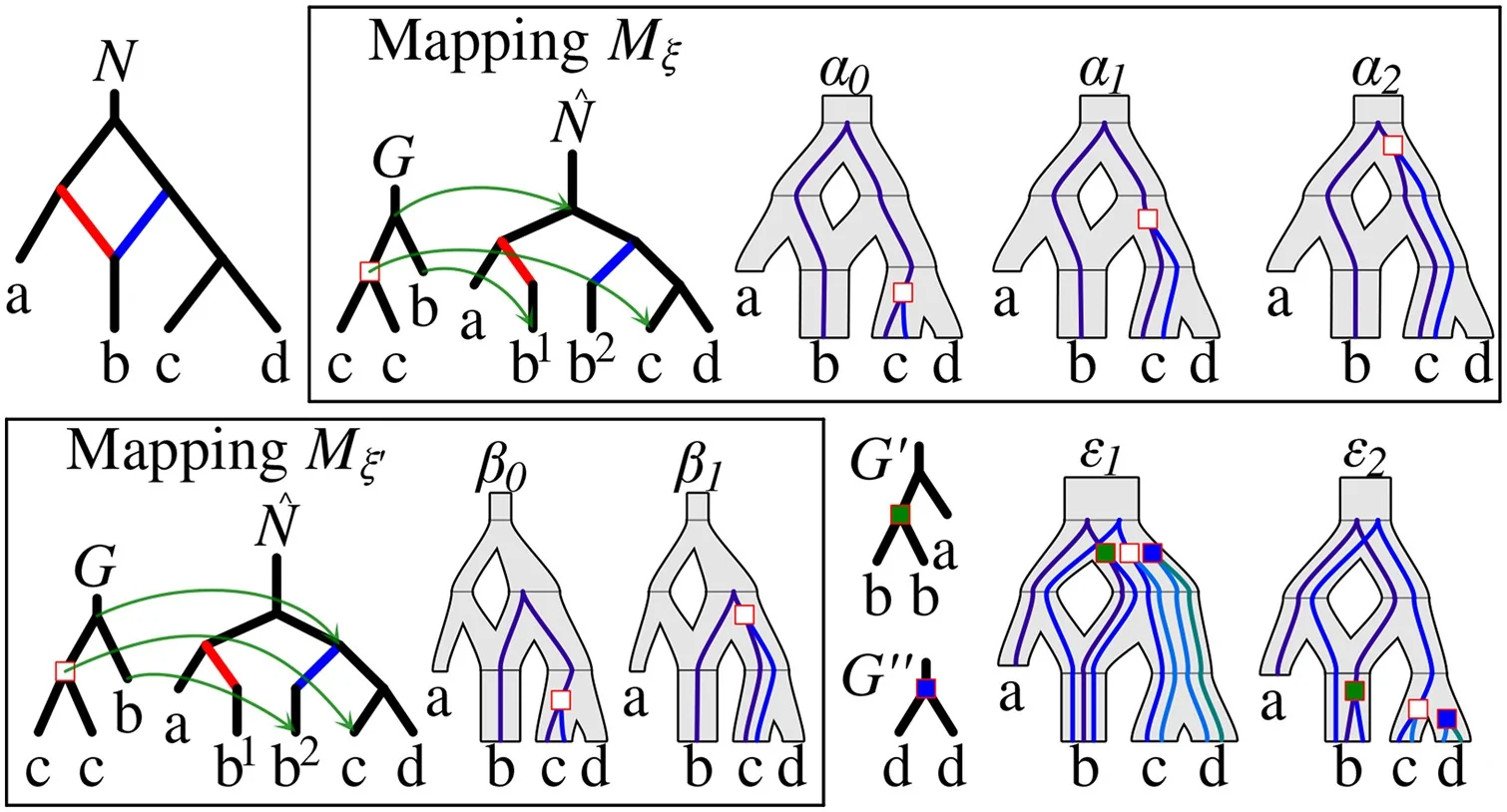
*Top-left*: A network *N* with one reticulation. *Framed Panels*: A gene tree *G*, an unfolded network $\hat{N}$ obtained from *N*, and two possible scenarios ξ and $\xi^{\prime }$. A scenario ξ routes gene tree leaves through one parent edge of the reticulation node, while $\xi^{\prime }$ routes them through the other, yielding two distinct histories. There are two lca-mappings $M_{\xi }$ and $M_{\xi^{\prime }}$ and five valid mappings $\alpha_{0}-\alpha_{2}$, $\beta_{0}$, and $\beta_{1}$ shown here as embeddings with duplications (squares). Embeddings $\alpha_{0}$ and $\beta_{0}$ correspond to $M_{\xi }$ and $M_{\xi^{\prime }}$. *Bottom-right*: two alternative episode clusterings $\epsilon_{1}$ and $\epsilon_{2}$ arising from gene trees *G*, $G^{\prime }$ and $G^{\prime \prime }$. In $\epsilon_{1}$, one episode at the root’s right child groups three duplications at a single network node, whereas $\epsilon_{2}$ assigns each duplication to a separate episode at leaves *b*, *c* and *d* as singletons. Our objective is to minimize the number of episode locations, so $\epsilon_{1}$ is preferred over $\epsilon_{2}$. Note that in embeddings, duplications are placed on the edges directly above their mapped nodes, reflecting that these events precede the corresponding speciation (or leaf) in evolutionary time.

For a network *N*, and, for each i=0,1,…,|R(N)| we define a pair $\left(N_{i},\pi_{i}\right)$ as follows. Let $N_{0}=N$ and $\pi_{0}$ be the identity function on V(N). Then, $\left(N_{i+1},\pi_{i+1}\right)$ is obtained from $\left(N_{i},\pi_{i}\right)$ by the unfolding operation: (1) pick a reticulation $p\in R(N_{i})$ such that no other reticulation is visible from *p*. Let $N_{i}^{p}$ be the subtree of $N_{i}$ rooted at *p* and let *u* denote an arbitrary parent of *p*; (2) copy $N_{i}^{p}$, (3) remove the edge (u,p) and add a new edge from *u* to the root of the copy of $N_{i}^{p}$. For $s\in V(N_{i+1})$ let $\pi_{i+1}(s):=\pi_{i}(s)$ if $s\in V(N_{i})$ and $\pi_{i+1}(s):=\pi_{i}(t)$, if *s* is a copy of a node $t\in V(N_{i})$. See $\hat{N}$ in Fig. 1.

$N_{\left|R(N)\right|}$ is called the *unfolded network* of *N* and denoted $\hat{N}$. Also, by π we denote $\pi_{\left|R(N)\right|}$. In other words, π is the projection of unfolded network nodes to the source nodes in *N*.

It follows from (Wawerka *et al.* 2022) that the unfolded network $\hat{N}$ of *N* is a semi-binary tree. There is a one-to-one correspondence between root-leaf paths in *N* and root-leaf paths in $\hat{N}$ established by π: if $P=p_{1},p_{2},\ldots ,p_{m}$ is a root-leaf path in $\hat{N}$, then $\pi (P)=\pi (p_{1}),\pi (p_{2}),\ldots ,\pi (p_{m})$ is the corresponding root-leaf path in *N*. Conversely, every root-leaf path in *N* arises in this way: each traversal of a reticulation in *N* corresponds to choosing exactly one of the two copies created during unfolding, so π restricted to root-leaf paths is bijective.

Note that the size of $\hat{N}$ grows exponentially with the size of R(N). It will become evident later that our algorithms do not use unfolded networks directly.

For a gene tree *G* over a taxa set *Y* from a network *N*, a *scenario* is a function $\xi :L(G)\to L(\hat{N})$ such that for each l∈L(G), Λ(l)=Λ(ξ(l)). An *lca-mapping in a scenario* ξ is a function $M_{\xi }:V(G)\to V(\hat{N})$ that extends ξ:


$$\begin{array}{c} M_{\xi }(g)=\{\begin{array}{cc} \xi (g) & if\;\;g\in L(G),\left(1\right)\\ \mathrm{lca}(M_{\xi }(g^{\prime }),\;M_{\xi }(g^{\prime \prime })) & \mathrm{otherwise},\left(2\right) \end{array} \end{array}$$

where lca(x,y) is the least common ancestor of *x* and *y*. A scenario can be visualized as an embedding of *G* into *N* (see Fig. 1). An internal node g∈V(G) is a *duplication* in a scenario ξ, or ξ*-duplication*, if $M_{\xi }(g)=M_{\xi }(a)$ for a child *a* of *g*. The remaining internal nodes are called ξ*-speciations*.

### 2.2 Episode clustering problems

We now formalize the model of duplication episodes for phylogenetic networks. Unlike the tree-based episode clustering model, our network-based approach must account for the multiplicity of evolutionary scenarios induced by reticulation nodes. The model admits all biologically plausible evolutionary scenarios with minimal duplication events.

In phylogenetic networks, episode clustering requires careful handling of the interplay between scenario selection (choosing paths through reticulations) and duplication assignment (determining episode locations). The model of gene duplication episodes allows relocating a gene duplication from its lca-mapping node to one of its ancestors, subject to additional constraints that preserve both biological soundness and scenario consistency. For a gene tree *G* over a set of taxa from a network *N*, a mapping $F:V(G)\to V(\hat{N})$ is *valid*, if there is a scenario ξ between *G* and *N* such that

- *(Time consistency)* F(a) ≼ F(b) if a ≼ b;
- *(Fixed speciations)* $F(a)=M_{\xi }(a)$ for any ξ-speciation or leaf *a*;
- *(Raised duplications)* $F(a)≽M_{\xi }(a)$ for any ξ-duplication *a*; duplications may move strictly upward;
- *(Duplication precedes speciation)* $F(a)≺M_{\xi }(b)$ for any ξ-speciation *b* with a≺b; raised duplications cannot cross a fixed speciation, preserving the count of ξ-duplications.

Note that if *F* is valid on ξ, then $F{|}_{L(G)}=\xi$. Therefore, if a valid *F* on ξ is given, we often use the term *F*-duplication (or *F*-speciation) instead of ξ-duplication (ξ-speciation, respectively). Additionally, *F*-duplications *g* satisfying $F(g)=M_{\xi }(g)$ are called *lca-duplications* (when the context *F* is clear).

In practice, we are interested in locations of events in the original network *N*, rather than $\hat{N}$. Therefore, let $F^{*}:V(G)\to V(N)$ be the mapping $F^{*}(g)=\pi (F(g))$ that allows a direct embedding of a gene tree into the network. We also write that a node g∈V(G) is *assigned* to s∈V(N) if $F^{*}(g)=s$. By Dup(F)⊆V(G), we denote the set of all *F*-duplications, while by Epi(F)⊆V(N) we denote the set of all *F*-duplication locations in the network *N*, defined as


$$\mathrm{Epi}(F)=\{F^{*}(g):g\in \mathrm{Dup}(F)\}.$$

Figure 1 provides an example of valid mappings that define an evolutionary scenario that can be represented as a tree with additional decoration of nodes. For details on the modelling of evolutionary scenarios, refer to (Górecki and Tiuryn 2006).

Assume that $F_{i}:G_{i}\to \hat{N}$ is a valid mapping between a gene tree $G_{i}$ over a set of taxa from a network *N*, for every i∈{1,2,…,n}. Every element in $\underset{i}{\cup }\mathrm{Epi}(F_{i})$ denotes a network location to which one or more gene duplications (across any number of families) have been assigned. We call these nodes *duplication episodes* or simply *episodes*; the signal of interest is the number of distinct episode locations, not the size of any individual episode—isolated single-gene duplications are episodes too, just not typically indicative of large-scale events.

**Problem 1** (Network Episode Clustering, NetEC). *Given a phylogenetic network N and a collection of rooted gene trees* $G_{1},G_{2},\ldots ,G_{k}$  *over the set of taxa present in N. Compute the minimum number of duplication episodes, denoted by* $\mathrm{EC}(G_{1},G_{2},\ldots ,G_{k},N)$*, in the set of all valid mappings* $F_{1},F_{2},\ldots ,F_{k}$  *between* $G_{i}$  *and* $\hat{N}$*, respectively.*

NetEC is solvable in linear time when *N* is a tree (Paszek and Górecki 2018b); its complexity for networks remains open.

## 3 Methods

Our approach to NetEC proceeds in three stages. First, we develop a constrained feasibility test that determines whether a given set of network nodes can serve as episode locations for a single gene tree. Second, we extend this test to compute the minimum episode clustering for a single gene tree. Third, we generalize to multiple gene trees. The key insight enabling our approach is the observation that while a phylogenetic network may encode exponentially many scenarios, we can efficiently reason about episode feasibility by working directly on the network structure.

### 3.1 Net-Episode feasibility

We start with a fundamental constrained problem. Given a gene tree and a phylogenetic network, we ask whether there exists a scenario and a corresponding valid mapping such that the set of duplication episodes is contained in a given fixed set of candidate episode locations in the network.

**Problem 2** (Net-Episode Feasibility). *Given a gene tree G over a set of taxa from a network N and* X⊆V(N)*. Does there exist a valid mapping F from G to* $\hat{N}$  *such that* Epi(F)⊆X*?*

If a gene tree *G* satisfies the above property, we call *G X-feasible* with respect to *N*. If the context is clear, we omit the reference to *X*. Reticulations can be excluded from episode locations: any duplication assigned to a reticulation is indistinguishable from one assigned to its unique child, so it can always be reassigned downward without changing the episode count. We therefore assume w.l.o.g. that X⊆V(N)∖R(N).

A challenge in network reconciliation is that duplications may be constrained to episodes not in the candidate set *X*. Still, these constraints may only become apparent when traversing upward in the network toward the root. To handle this uncertainty, we employ Łukasiewicz’s Three-Valued Logic ${Ł}_{3}$ (Łukasiewicz 1970), which extends Boolean logic with an Unknown value representing situations where episode assignment is deferred to higher levels of the network. This three-valued logic has constants True, False, and Unknown ordered linearly as False<Unknown<True, with binary operators ∨ (disjunction, max) and ∧ (conjunction, min). The unary operators are defined as: L (certainty operator), where L(x)=True if x=True and L(x)=False otherwise; and M (possibility operator), where M(x)=False if x=False and M(x)=True otherwise.

For a node *v* of a tree *T*, by T|v we denote the subtree of *T* rooted at *v*. Recall that $s^{\prime }$ and $s^{\prime \prime }$ denote the children of *s*, with only $s^{\prime }$ defined for reticulations, and similarly for *g*. To simplify the notation, we assume that the set X⊆V(N) is fixed.

The dynamic programming formulas to solve the feasibility problem are given below; here ch(s) denotes the children of *s* (empty for leaves). For g∈V(G) and s∈V(N)∖R(N):


$$\begin{array}{c} \delta (g,s)=\{\begin{array}{cc} \delta^{*}(g,s) & g\;\;\text{internal},\;\;s\in X,\left(3\right)\\ \delta^{*}(g,s)\wedge \text{Unknown} & g\;\;\text{internal},\;\;s\notin X,\left(4\right)\\ \text{False} & \text{otherwise},\left(5\right) \end{array} \end{array}$$

(6) $$\delta^{*}(g,s)=\epsilon (g^{\prime },s)\wedge \delta^{↓}(g^{\prime \prime },s)\vee \epsilon (g^{\prime \prime },s)\wedge \delta^{↓}(g^{\prime },s),$$

$$\delta^{↓(}g,s)=\epsilon (g,s)\vee \{\begin{array}{c} \underset{c\in ch(s)}{\vee }M\;\;\delta^{↓}(g,c)s\;\text{non-leaf},s\in X,\left(7\right)\\ \underset{c\in ch(s)}{\vee }\delta^{↓(}g,c)\text{otherwise},\left(8\right) \end{array}$$

$$\begin{array}{c} \sigma (g,s)=\{\begin{array}{c} L\;(\delta^{↓(}g^{\prime },s^{\prime })\wedge \delta^{↓(}g^{\prime \prime },s^{\prime \prime })\\ \vee \delta^{↓(}g^{\prime },s^{\prime \prime })\wedge \delta^{↓(}g^{\prime \prime },s^{\prime }))\;\;\;g\;\;\;\text{internal},\;s\;\;\;\text{tree-node},\left(9\right)\\ \text{True}g\;\;\;\text{leaf}\;\;\;\text{labelled}\;\;\;s,\left(10\right)\\ \text{False}\text{otherwise},\left(11\right) \end{array} \end{array}$$

(12) $$\text{For}\;\;s\in R(N):f(g,s)=f(g,s^{\prime })\;\text{for}\;f\in \{\delta ,\delta^{*},\delta^{↓},\sigma \}.\text{Auxiliary function}:\epsilon (g,s)=\sigma (g,s)\vee \delta (g,s).$$

For a gene tree *G* over *N* and a node g∈V(G), let $F:V(G|g)\to V(\hat{N})$ be a valid mapping. We say that *F* is *feasible* for (g,s,X) if and only if $F^{*}(g)\;≼\;s$ and Epi(F)⊆X. Feasible mappings represent episode scenarios that correspond to partial solutions to the instance of Net-Episode Feasibility that have all duplications present in *X*. We say that an *F*-duplication *d* in a gene tree *T* is *upper* if the path from *d* to *g* consists of *F*-duplications. The set of all upper *F*-duplications we denote ∪Dup(F)⊆Dup(F) and their locations in *N* we denote by ∪Epi(F)⊆Epi(F). We write that $F:V(G|g)\to V(\hat{N})$ is *weakly feasible* for (g,s,X) if and only if $F^{*}(g)\;≼\;s$, g∈∪Dup(F) and Epi(F)∖∪Epi(F)⊆X.

In a weakly feasible mapping, only non-upper duplications in *G|g* are required to land in *X*; upper duplications are deferred and will be absorbed by some ancestor episode $\tilde{s}\in X$ with $s≺\tilde{s}$, chosen later in the recursion. This deferral is what the value Unknown represents in the returns of $\delta^{↓}(g,s)$ and δ(g,s).

Informally, the meaning of DP formulas can be understood as follows: δ(g,s) is True if there is a feasible mapping *F* for (g,s,X) such that s∈X, *g* is an *F*-duplication assigned to s∈X, and all duplications are assigned to the episodes from *X*. Similarly, σ(g,s) is True if there is a feasible mapping *F*, where $F^{*}(g)=s$ and *g* is an *F*-speciation or a leaf. Next, δ(g,s) is Unknown if there is no feasible mapping for (g,s,X), however, there is a weakly feasible mapping *F* for (g,s,X), where *g* is an *F*-duplication assigned to s ∉ X, and all non-upper duplications from *G|g* are assigned to the episodes from *X*. Note that σ(g,s) cannot be Unknown since speciation nodes are fixed. Moving on, $\delta^{↓}(g,s)$ is True if there is a feasible mapping for (g,s,X), where all duplications are assigned to the episodes from *X*. Lastly, $\delta^{↓}(g,s)$ is Unknown if the condition for $\delta^{↓}(g,s)=\mathrm{True}$ is not met. However, there is a weakly feasible mapping for (g,s,X).

To solve Net-Episode Feasibility, we apply $\delta^{↓}$ on the roots.

**Theorem 1** (Correctness). *Given a gene tree G over a network N and* X⊆V(N)∖R(N)*. G is X-feasible if and only if*  $\delta^{↓}(\mathrm{root}(G),\mathrm{root}(N))$  *is* True.

Finally, the time and space complexity of solving Episode Feasibility by the DP algorithm is O(|V(G)|·|V(N)|).

For feasible instances, the backtracking can identify the subset of nodes from *X* that contribute to the optimal solution by quantifying the number of duplications (i.e. episode sizes for each episode). These duplication counts provide insights into the significance of each inferred episode.

See Appendix for the proofs.

### 3.2 Solution for a single gene tree and the general case

First, we describe the main algorithm to solve NetEC for instances with a single gene tree.

Algorithm 1 extends (Górecki *et al.* 2024) by first identifying *fixed episodes* (nodes present in every solution) through testing feasibility with each node excluded; if removal makes the instance infeasible, that node is fixed. The main loop then searches for a set C⊆V(N)∖Φ of size b−|Φ|−1 such that *G* is (C∪Φ)-feasible, updates *b* via DP backtracking, and terminates when no such *C* exists.

**Algorithm 1** Solution to NetEC with a single gene tree

**Require:** A gene tree *G* over a network *N*

**Ensure:**  EC(G,N)

1: Φ←∅       ▹ Init: the set of fixed episodes Φ

2: **for**  *v* in V(N)∖R(N)  **do** ▹ Identify fixed episodes

3:  **if** there is no feasible mapping for V(N)∖{v}  **then** 

4:   Add *v* to Φ (*v* is a fixed episode)

5: b←|V(N)∖R(N)| ▹ The initial maximal number of episodes

6: **while**  there is C⊆V(N)∖Φ of the size b−|Φ|−1 and *G* is  (C∪Φ) -feasible **do**         ▹ The main loop

7:  b← the EC cost by backtracked DP from Eqs. (3)–(11).

8: Optional backtracking: compute the episode sizes by  counting duplications at given episode s∈X when (3) is  reached in DP.

9: **return** *b*

The correctness of Alg. 1 follows from the fact that if there is no set *X* of size b−1 such that *G* is *X*-feasible, then there is no set of any size smaller than *b* satisfying the property. Since *b* represents the number of episodes from some valid mapping, it is also minimal. Thus, Alg. 1 returns b=EC(G,N).

The algorithm’s worst-case time complexity is $n^{2}m+\sum_{k=0}^{n-f-1}\left(\begin{array}{c} n-f\\ k \end{array}\right)nm=O(nm\cdot 2^{n-f})$, where *f* is the size of the set of fixed episodes (f=|Φ|), *n* denotes the number of nodes in *N*, and *m* denotes the number of nodes in *G*. Despite the exponential time complexity, in our experiments on both simulated and empirical data, we were able to compute exact solutions after only a few executions of the main loop.

To identify the optimal solution within the main loop, enumerating all possible combinations of size b−f−1 from the set of episode candidates V(N)∖Φ may be time-consuming for larger instances. To address this issue, we propose a heuristic approach that randomly samples combinations of size b−f−1 if $\left(\begin{array}{c} n-f\\ b-f-1 \end{array}\right)$ is large, similarly to our previous solution from (Górecki *et al.* 2024). In our experiments, the heuristic mode was not reached.

To solve NetEC in a general case, we transform the problem to a single gene tree case. Given a collection of gene trees $G_{1},G_{2},\ldots ,G_{k}$ and a network *N*. Let ω be a new species, called *outgroup*, not present in *N*. We first add the outgroup to every input tree. Throughout, (T,ω) denotes the rooted tree obtained by joining a tree *T* and a single new leaf labelled by ω at a fresh root. Let $G_{1}^{\omega }=(G_{1},\omega )$ and $G_{i}^{\omega }=((G_{i},\omega ),G_{i-1}^{\omega })$, for i>1. Let $N^{\omega }$ be a network obtained from *N* by inserting a new root and a leaf labelled by the outgroup and connecting the new root with the leaf and the root of *N*. Then, by ω-NetEC we define the problem NetEC with a single gene tree.

**Lemma 2.**  *Given at least two gene trees* $G_{1},G_{2},\ldots ,G_{k}$  *and a network N such that* ω ∉ L(N)*. Then*, X⊆V(N)  *is the set of episodes that yields the solution of NetEC for* $G_{1},G_{2},\ldots ,G_{k}$  *and N if and only if* $X\cup \{\mathrm{root}(N^{\omega })\}$  *is the set of episodes that yields the solution to the instance* $G_{k}^{\omega }$  *and* $N^{\omega }$  *of* ω*-NetEC.*

For *k* gene trees, the construction merges them into a single tree of size O(k+M), where $M=\sum_{i=1}^{k}|V(G_{i})|$, so the overall time complexity becomes $O\left(n(k+M)2^{n}\right)$ where n=|V(N)|.

### 3.3 Post-evaluation: extended episodes analysis

**Figure 2:**
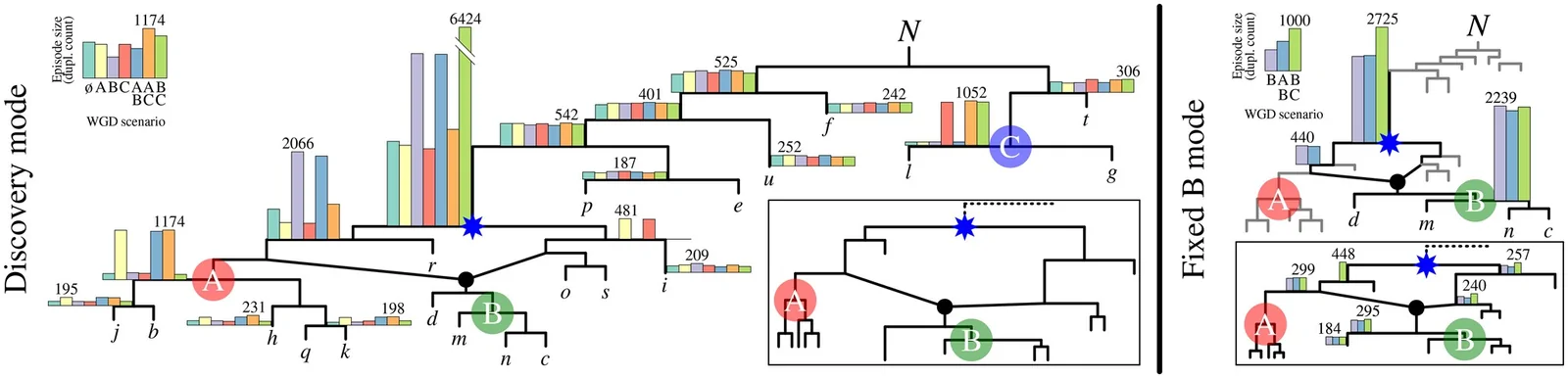
Inference of duplication episodes from seven simulated WGD scenarios on phylogenetic network *N* (one reticulation node, black circle). Nodes *A*, *B*, *C* indicate simulated WGD positions across seven scenarios: ∅, *A*, *B*, *C*, *AB*, *AC*, *BC*. Histograms show inferred episode sizes (number of gene duplications); values above indicate maximum frequency; histograms with all values below 150 are omitted. *Left*: Discovery mode results. *Middle frame*: Extended analysis (Alg. 2) reveals node *B* as an additional high-support episode in scenarios *B*, *AB*, and *BC*. *Right*: Results after fixing node *B* as an extended episode in scenarios *B*, *AB*, and *BC*. *Right frame*: Extended analysis confirms no further episode candidates. Note that branch lengths of *N* are adjusted for clarity across all visualizations and do not reflect its ultrametric structure.

Our experiments revealed that duplications located below a reticulation node may cluster at the *lowest stable ancestor* (LSA) of the reticulation (i.e. the lowest common ancestor of all parents) or higher nodes. This artefact results from the flexible mapping model, which permits scenarios utilizing both reticulation edges, combined with the episode minimization objective. The left part of Fig. 2 illustrates this: duplications from WGD events at *B* are moved to the LSA (marked by a star). Alg. 2 addresses this by iteratively testing non-episode nodes in post-order and adding those whose episode size exceeds a threshold based on the average episode size ($\bar{e}$). Processing nodes bottom-up ensures that the lowest high-signal candidates are identified first, preventing their duplications from being absorbed by higher-level episodes.

**Algorithm 2** Extended episodes analysis

**Require**: A set of gene trees over a network *N*, τ =2 (default).

**Ensure**: The set of episodes with extended episodes

1: Merge gene trees into a single tree (see Lemma 2).

2: Compute the set of episodes *Q* using Alg. 1.

3: **for**  s∈V(N)∖Q in post-order **do** 

4:  Compute the episode size of *s* using DP with X=Q∪{s}

5:  **if** the size of episode *s* exceeds $\tau \cdot \bar{e}$  **then** 

6:   Add *s* to *Q*    ▹  *s* becomes an extended episode

7: **return** *Q*

## 4 Results

We evaluate NetEC on simulated data with known WGD ground truth and on an empirical Pandanales dataset comprising over 29 000 gene trees on a network with two reticulations. All experiments were performed using the NetEC tool (https://github.com/ppgorecki/netec); input data, scripts, and parameter settings to reproduce the experiments are available in the same repository. The tool operates in two modes: *discovery* mode for direct inference and *verification* mode, in which users specify candidate WGD locations. Dataset preparation is described at the start of each subsection.

### 4.1 Simulated data

#### 4.1.1 Data preparation

We sampled ultrametric tree-child networks of height $1.8\times 10^{9}$ years based on species trees with 20 leaves and one reticulation event using (Rutecka *et al.* 2024), selecting a network *N* where the reticulation connects temporally proximate lineages. *N* is depicted in Fig. 2 (reticulation marked as a black circle). Each simulated dataset corresponds to one of seven WGD scenarios (∅, *A*, *B*, *C*, *AB*, *AC*, *BC*); the scenario name lists the species-tree nodes at which a WGD was injected during gene-tree simulation, and these node sets form the ground truth against which inferred episodes are compared.

We identified three candidate locations for WGD events, denoted *A*, *B*, and *C*. Location *A* is positioned close to but independent of the hybridization event, representing WGD that occurs without direct relationship to the hybridization process. Location *B* is positioned below the reticulation in *N* and therefore appears twice in $\hat{N}$, once on each descendant branch following hybridization. Location *C* is evolutionarily distant, positioned on a branch unrelated to the hybridization event.

We analyzed seven scenarios in total: no WGD (∅), single WGDs at locations *A*, *B*, or *C*, and all pairwise combinations (*AB*, *AC*, *BC*). Whole-genome duplication events were simulated following (Paszek *et al.* 2021). For a given node *v* in tree $\hat{N}$, a WGD event at *v* was modelled by replacing the corresponding subtree $\hat{N}|v$ with its duplicated copy $\left(\hat{N}|v,\hat{N}|v\right)$. When the duplication event occurred below a reticulation, this substitution was applied symmetrically to both unfolded parts of the network. Consequently, we generated seven species trees from the unfolded network $\hat{N}$ for each WGD scenario.

Our simulation study consists of several phases to infer gene trees under biologically realistic conditions. A total of 1000 replicates were generated for each of seven species trees, yielding 7 000 gene trees in total. True gene trees were simulated using SimPhy (Mallo *et al.* 2016) under a multilocus coalescent model incorporating incomplete lineage sorting and gene duplication/loss. We employed a duplication/loss rate of $2\times 10^{-10}$ events per year and an effective population size of $N_{e}=10^{7}$. Gene tree heights were drawn from a lognormal distribution (μ=1.5, σ=1). DNA sequences of 1000 bp were simulated along the true gene trees using AliSim (Minh *et al.* 2020) under the GTR+Γ model. Model parameters were sampled from empirical Dirichlet priors following (Molloy and Warnow 2020). Insertions and deletions were simulated with rates of 0.03 and 0.09 per substitution, respectively, with lengths following a Zipfian distribution (exponent 1.7, maximum length 50). Sequences were aligned using MAFFT (Katoh and Standley 2013), and maximum likelihood trees were estimated from these inferred alignments using PhyML (Guindon *et al.* 2010) under the GTR+Γ model with estimated parameters. The unrooted trees were rooted by midpoint-plateau rooting implemented in URec (Górecki and Tiuryn 2007). All datasets were processed by the NetEC tool within 2 hours on a standard workstation.

#### 4.1.2 Results

The results of the discovery mode (Alg. 1) applied to the simulated datasets are depicted in Fig. 2, where inferred episode sizes are presented as histograms attached to corresponding nodes of *N*. Across the simulated WGD scenarios, fixed episodes accounted for 69–74% of network nodes across the seven scenarios, substantially reducing the combinatorial search performed in the main loop.

Our solution minimizes the *number* of duplication episode locations, not the episode sizes themselves. Because the simulation includes background single-gene duplications and incomplete lineage sorting (ILS), small non-zero episode sizes appear at many nodes even in the absence of any WGD. To distinguish WGD-driven episodes from background, we report any episode whose size exceeds $\tau \cdot \bar{e}$, where $\bar{e}$ is the mean episode size over non-leaf nodes and τ is a user-set multiplier (default τ=2, also used as threshold in Alg. 2, line 7). On our simulated data this rule recovers the planted WGD locations in scenarios *A*, *C*, *AC* directly, and *B*, *AB*, *BC* after extended analysis, without false positives.

For WGD scenarios *A*, *C*, and *AC*, NetEC tool successfully inferred the events in all experimental settings where they were simulated. These scenarios exhibited strong signals exceeding 1000 duplications, whereas in evolutionary scenarios lacking the corresponding WGD, the signal matched the background episode sizes of the null scenario (∅). WGD events at locations *A* and *C* were also correctly identified with significant episode sizes in scenarios *AB* and *BC*, respectively.

In contrast, the event at location *B* was not detected in scenarios *B*, *BC*, and *AB*. We observed that duplications from *B* were likely reassigned upward to the LSA node (marked by blue star in Fig. 2) and partially to the LSA’s left child. This upward-attraction phenomenon mirrors an analogous observation in tree-based unrestricted minimal episodes inference (Iersel *et al.* 2020), where optimal solutions tend to place episodes near the root; in that context the authors addressed it by changing the objective (minimizing the maximum episode depth along any root-to-leaf path), while here we mitigate it through post-hoc extended episode analysis (Alg. 2), which preserves the simpler episode-count objective.

We then performed extended episode inference by examining additional candidates among non-episode nodes (Alg. 2). The results are summarized in the bottom-right panel of Fig. 2, where the method identified node *B* as the only extended episode with high support (above 2000 duplications) in WGD scenarios *B*, *BC*, and *AB*. For the remaining scenarios, episode sizes were small, indicating that the inference was complete.

In the subsequent analysis, we employed NetEC tool with the extended episode at location *B* for the three WGD scenarios *B*, *BC*, and *AB*. The results are shown in the right side of Fig. 2, where the number of duplications at location *B* correctly indicates the simulated WGD event. We also observed that the number of duplications at the LSA node and its child were significantly reduced compared to the previous analysis.

Finally, performing extended episode analysis on this inference did not identify any additional episode candidates (see bottom-right panel of Fig. 2).

### 4.2 Empirical evaluation: Pandanales

#### 4.2.1 Data preparation

**Figure 3:**
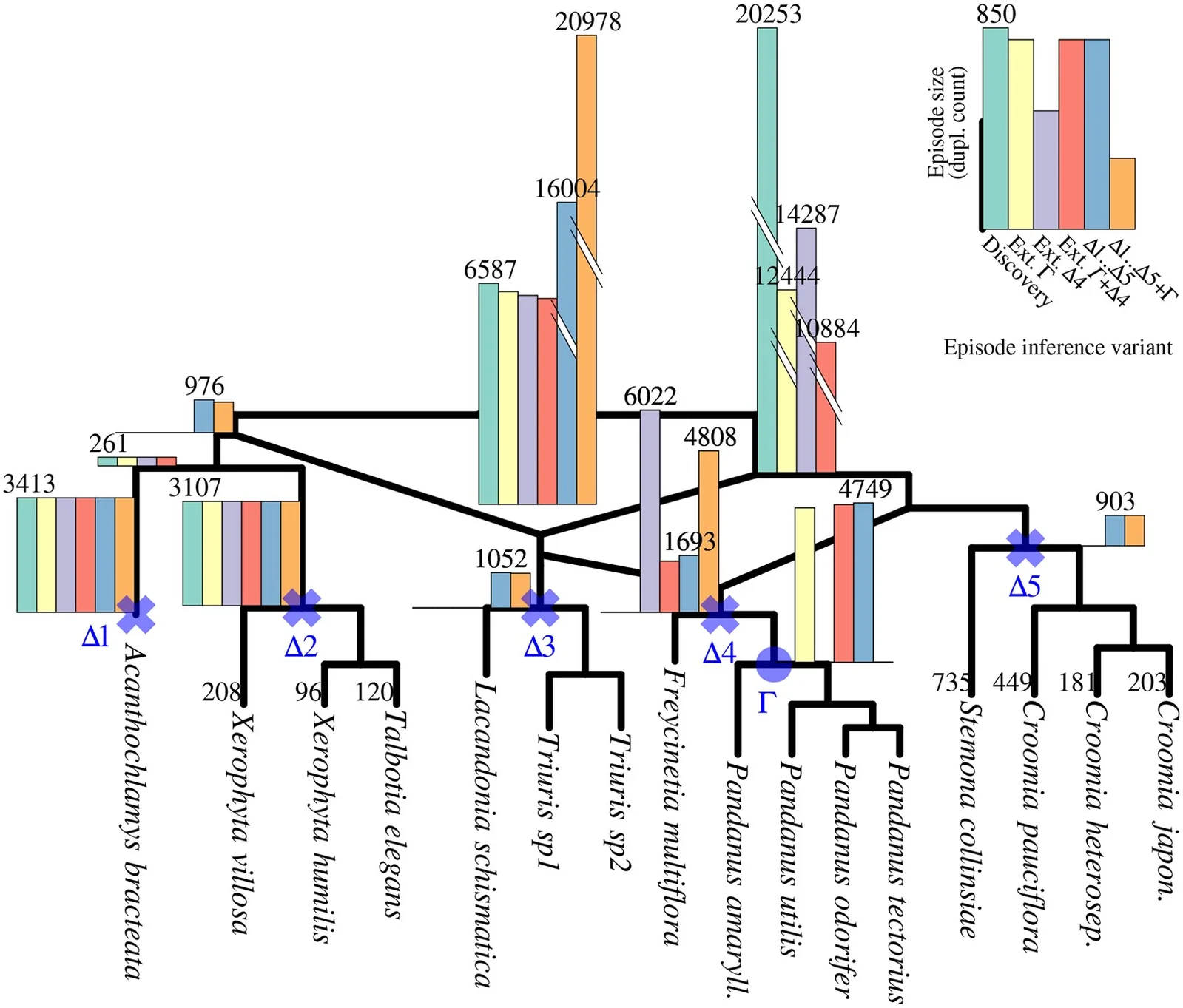
Episode inference on empirical Pandanales gene trees on a phylogenetic network with two reticulations. The network follows (Shi and He 2025) with five hypothesized WGD events (Δ1-Δ5, blue crosses) and an additional candidate node Γ (blue circle). Bar charts at nodes display episode sizes under six inference variants: discovery mode alone, extended analysis forcing Γ, extended analysis forcing Δ4, combined extended analysis (Γ+Δ4), hypothesis-driven mode with all five published WGDs (Δ1-Δ5), and hypothesis-driven mode including Γ (Δ1-Δ5+Γ). Numbers above bars indicate episode sizes, while the numbers near nodes without histograms represent equal duplication count obtained in all six inferences. Results show strong support for Δ1, Δ2, and either Δ4 or Γ (which absorbs signal from Δ4 when included), with moderate support for Δ5. Deep ancestral nodes accumulate duplications due to upward clustering permitted by reticulation-induced scenario flexibility.

We investigated the placement and support of WGD events in a reticulate evolutionary history of Pandanales. Starting from the Pandanales species tree, we extended it to a phylogenetic network by introducing two reticulation events inferred by Shi and He (2025) using HyDe-based gene-flow tests. As the duplication signal, we used 29 453 gene trees reconstructed from sequence data available in (Shi and He 2025); sequences were processed and partitioned into families following the pipeline described in that study, with gene trees inferred using IQ-TREE under the GTR+Γ model. We performed two experiments: a discovery run and a hypothesis-driven run in which the five published WGD nodes (Δ1–Δ5, Fig. 3) were provided as user-specified episodes to test whether duplications concentrate at those locations. The analysis by NetEC tool, including all inference variants, completed within 12 hours.

#### 4.2.2 Results

In discovery mode (Fig. 3; first bars), support concentrates at deep ancestral nodes (right child of root: 20 253; root: 6587), reflecting upward clustering below reticulations. Among the published WGDs, Δ1 and Δ2 are directly recovered (3413 and 3107), while Δ3–Δ5 are not. Extended analysis identified only two high-signal candidates: Γ (4599) and Δ4 (6022). Forcing episodes at these nodes individually and jointly (Fig. 3) reveals that Δ4 absorbs more duplications alone, but Γ captures the larger local fraction when both are included (4696 vs. 1524 at Δ4). This illustrates that extended episodes are best applied iteratively: inserting a high-signal episode and recomputing reveals whether a residual peak warrants an additional episode.

In hypothesis-driven mode, inserting Δ1-Δ5 as user-specified episodes yields strong support for Δ1, Δ2, and Δ4, moderate for Δ5, and weaker for Δ3, with a large fraction remaining at deep nodes. Extended analysis further suggests Γ as an additional episode (4749), reducing Δ4 to 1693 and indicating Γ as a more appropriate WGD location.

Taken together, minimizing episode count can favour deeper placements when reticulation allows duplications to shift upward, and extended analysis is essential for revealing biologically relevant WGD candidates.

## 5 Discussion and conclusions

Here, we propose the first algorithmic framework for identifying duplication episodes in phylogenetic networks. Experiments demonstrated that our approach accurately recovers genomic duplication events in the presence of reticulation, though with important caveats. Events on lineages not directly involved in hybridization are consistently detected with a strong signal. In contrast, WGD events below reticulation nodes require the extended episode procedure for reliable detection, as the flexible mapping model allows their duplications to be reassigned to ancestral positions. The extended analysis should therefore be considered a standard component of the inference pipeline. While the feasibility test runs in polynomial time, the overall algorithm has exponential worst-case complexity. Larger networks may require heuristic sampling, yielding upper bounds rather than guaranteed optima. The model assumes correctly rooted gene trees with known leaf-to-taxa mappings, does not explicitly handle gene losses or synteny, and incomplete lineage sorting contributes to the background signal observed in the null scenario. NetEC also reports a per-episode duplication-count metric without a calibrated significance score, and the threshold τ is user-chosen; reliability could be quantified via bootstrap over gene-tree subsamples or permutation-based *P*value. When the input network has no reticulations, NetEC reduces to the tree-based method of (Górecki *et al.* 2024). Validation on a second reticulate dataset, establishing the complexity of NetEC, joint topology-episode estimation, and extension to time-calibrated networks are further directions.

## Author contributions

Paweł Górecki (Conceptualization, Formal analysis, Funding acquisition, Methodology, Software, Supervision, Visualization, Writing—original draft), Agnieszka Mykowiecka (Data curation [equal], Investigation [equal], Resources [equal], Validation [equal], Writing—review & editing [equal]), and Jarosław Paszek (Data curation [equal], Investigation [equal], Resources [equal], Validation [equal], Writing—review & editing [equal])

## Supplementary material

Supplementary material is available at *Bioinformatics* online.

## Supplementary Material

btag445_Supplementary_Data
