# Supplementary material for: Episode clustering in phylogenetic networks
Source: Bioinformatics. 2026 Aug 10;42(Suppl 2):btag445. doi: 10.1093/bioinformatics/btag445 (PMC13501289; doi:10.1093/bioinformatics/btag445)
Supplement: Supplementary file 1 [file btag445_supplementary_data.pdf]

Supplement to: Episode Clustering in Phylogenetic Networks

Paweł Górecki* Agnieszka Mykowiecka
Jarosław Paszek

Faculty of Mathematics, Informatics, and Mechanics,
University of Warsaw, Banacha 2, 02-097 Warsaw, Poland

Abstract

This document is the online supplement to 'Episode Clustering in Phylogenetic Networks'. It contains the full proofs and auxiliary lemmas omitted from the main paper. Cross-references such as Lemma 1, Eq. (1) point back to the main manuscript.

Keywords: Genomic Duplication, Duplication Episode, Gene Tree, Species Tree, Phylogenetic Network.

Appendix: DP correctness lemma

Lemma 1. *Given a gene tree G over a network N and $X \subseteq V(N) \setminus R(N)$. Let $g \in V(G)$, $s \in V(N)$ and $\dot{s} = s'$ if $s \in R(N)$, and $\dot{s} = s$, otherwise. Then,*

P1 $\delta(g, s) = \mathsf{True}$ *if and only if there is a feasible mapping F for $(g, \dot{s}, X)$ such that g is an lca-duplication assigned by F to $\dot{s}$.*

P2 $\delta(g, s) = \mathsf{Unknown}$ *if and only if there is no feasible mapping for $(g, \dot{s}, X)$, but there is a weakly feasible mapping F for $(g, \dot{s}, X)$ such that g is an lca-duplication assigned by F to $\dot{s}$.*

P3 $\sigma(g, s) = \mathsf{True}$ *if and only if there is a feasible mapping F for $(g, \dot{s}, X)$ such that g is a speciation or a leaf assigned by F to $\dot{s}$.*

P4 For any g and s, $\sigma(g, s) \neq \mathsf{Unknown}$.

P5 $\delta^{\downarrow}(g, s)$ *is* True *if and only if there is a feasible mapping for $(g, \dot{s}, X)$.*

P6 $\delta^{\downarrow}(g, s)$ *is* $\mathsf{Unknown}$ *if and only if there is no feasible mapping for $(g, \dot{s}, X)$, but there is a weakly feasible mapping for $(g, \dot{s}, X)$.*

The auxiliary functions ϵ and δ^* are treated as local (no separate arrays in implementation). If $\epsilon(g, s) = \mathsf{True}$, there is a feasible mapping F for (g, s, X) with $F^*(g) = s$; if $\epsilon(g, s) = \mathsf{Unknown}$, then $\sigma(g, s) = \mathsf{False}$ and $\delta(g, s) = \mathsf{Unknown}$, and g is still assigned to s. $\delta^*(g, s) = \mathsf{True}$ requires a feasible mapping where g is a duplication at s with at least one child also mapped to s (modelled by ϵ); $\delta^*(g, s)$ resembles $\delta(g, s)$ but constrains only non-upper duplications when $\mathsf{Unknown}$.

*Corresponding author: gorecki@mimuw.edu.pl

Preliminary properties

Before starting the main proof, we need several additional notions and properties.

To preserve validity of mappings when merging mappings from subtrees, we need an operation that shifts a mapping in unfolded network to a proper copy of a subtree in $\hat{N}$. For a valid mapping $F\colon V(G|g) \to V(\hat{N})$ such that $F(g) = v$ and $w \in \pi^{-1}(v)$, by $F_w\colon V(G|g) \to V(\hat{N})$ we denote a mapping defined using a top-down approach. Let $F_w(g) := w$. If x is a child of $y \preceq g$, then the path $p_1, p_2, \ldots, p_k$ from $p_1 = F(y)$ to $p_k = F(x)$ induces the path $P = \pi(p_1), \ldots, \pi(p_k)$ in N. If $F_w(y)$ is already determined, then $F_w(x)$ is the unique node in $\hat{N}$ such that the path from $F_w(y)$ to $F_w(x)$ in $\hat{N}$ induces the path equal to P in N. Note that $\pi(F) = \pi(F_w)$, however, the main property is stronger. It follows from the construction that both mappings induce the same paths in a network N.

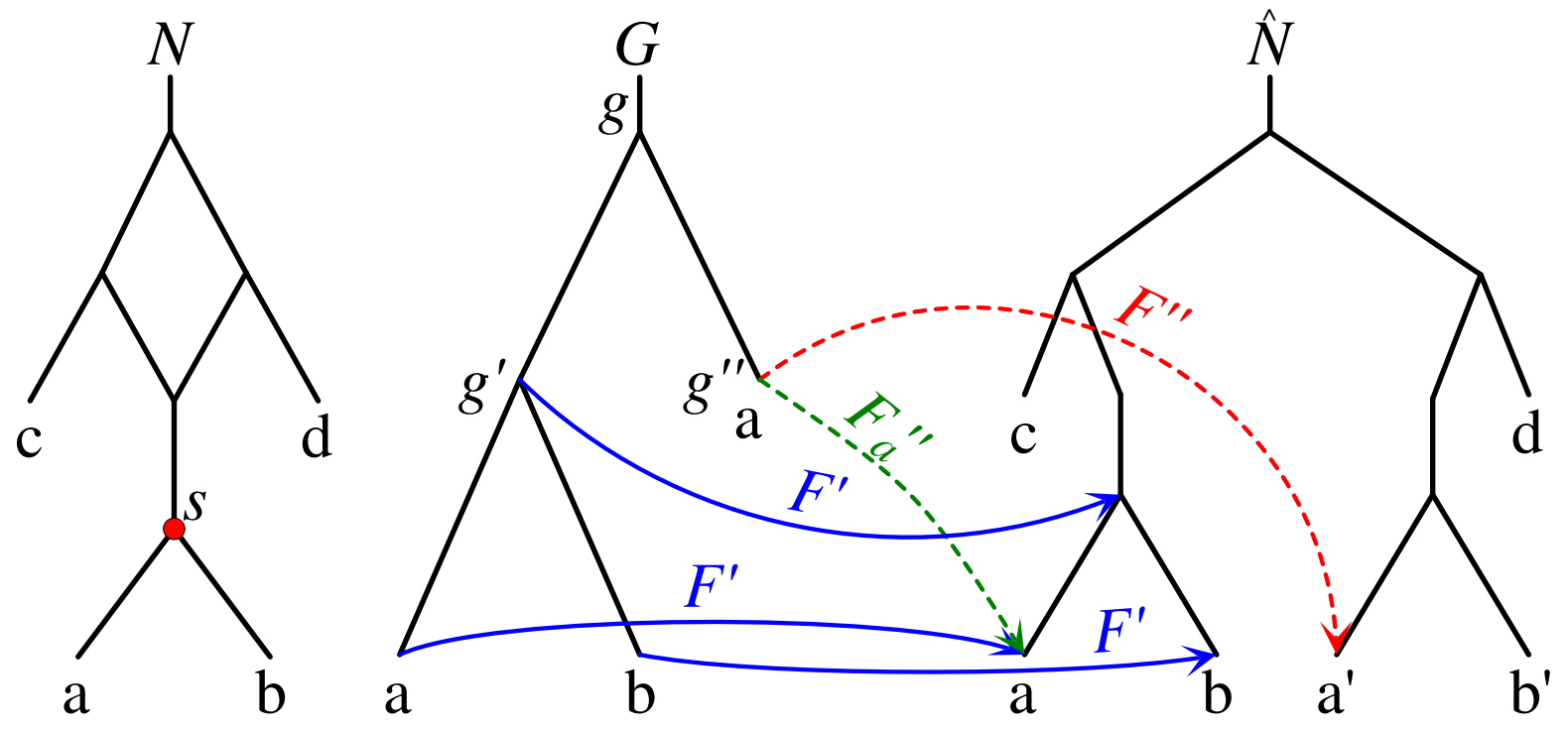

Figure 1: Network N and mappings F', F'' and F''_a. F' is valid for (g', s, X), while F'' and F''_a are valid for (g'', s, X), where $X = \{s\}$. Note that $F' \oplus F''$ is valid for (g, s, X), but F'' has to shifted (see F''_a) such that g'' maps to a (not a') in $\hat{N}$. Then, in $F' \oplus F''$, g is a duplication assigned to s. Without the shift, the resulting product of F' and F'' would map g to the root of $\hat{N}$.

Since functions are relations, we identify a function $f\colon A \to B$ with the sets of pairs $\{(x, f(x))\colon x \in A\}$. Let $F'\colon V(G|g') \to V(\hat{N})$ and $F''\colon V(G|g'') \to V(\hat{N})$ be valid mappings for (g', s, X) and (g'', s, X), respectively, such that $F'^{*}(g') \succeq F''^{*}(g'')$. Then, by $F' \oplus F''$ we denote the mapping $F'|_{V(G|g')\setminus \mathsf{UDup}(F')} \cup F''_w|_{V(G|g'')\setminus \mathsf{UDup}(F'')} \cup \{(g, v)\} \cup (\mathsf{UDup}(F') \cup \mathsf{UDup}(F'')) \times \{v\}$ where $F'(g) = v$ and $F''_w\colon V(G|g'') \to V(\hat{N})$ is the copy of F'' shifted to be present below v in $\hat{N}$, where w is a node such that $\pi(w) = F''^{*}(g'')$ and $v \succeq w$. Such a node exists since $F'^{*}(g') \succeq F''^{*}(g'')$.

Lemma 2. *Let $F'\colon V(G|g') \to V(\hat{N})$ and $F''\colon V(G|g'') \to V(\hat{N})$ be valid mappings for (g', s, X) and (g'', s, X), respectively, such that $F'^{*}(g') \succeq F''^{*}(g'')$. Then, $F' \oplus F''$ is valid for a (g, s, X) with g being a duplication assigned to s.*

Note that, without the shift operation, g may be assigned to a node above s. See example in Fig. 1.

Similarly, to $F' \oplus F''$, we define a mapping $F' \otimes F''\colon V(G|g) \to V(\hat{N})$. Here, F' is a mapping feasible for (g', s', X), F'' is a mapping feasible for (g'', s'', X), and s' and s'' are the children of s in N. In such a case the mapping is defined as $F' \cup F''_w \cup \{(g, v)\}$, where v is the parent of $F(g')$ in $\hat{N}$ (note that $\pi(v) = s$) and w is defined as in the previous paragraph. The proof of the next Lemma follows immediately from the definition of the mapping.

Lemma 3. *Let F' and F'' be mappings feasible for (g', s', X) and (g'', s'', X), respectively, where s' and s'' are the children of s in N. Then, $F' \otimes F''$ is valid for a gene tree $G|g$, where g is a speciation assigned to s.*

Proof. It follows from the definition of the mapping. $\square$

The main proof of Lemma 1 (DP Correctness)

The proof of Lemma 1 is by induction on the structure of G and N. The base of induction is when $g \in L(G)$ and $s \in L(N)$, for which all properties are easy to verify.

Inductive assumption: For every x, y such that $g \succ x$, and $s \succeq y$ or $g \succeq x$ and $s \succ y$, P1-P6 are satisfied. *Inductive hypothesis:* For g and s, where at least one of g and s is not a leaf, P1-P6 are satisfied.

First, if $s \in R(N)$ then the properties follow immediately from the inductive assumption for g and s', and identities (12). Therefore, in the next part, we assume that s is not a reticulation, and we prove the properties using s instead of $\dot{s}$.

We start with several properties.

(A1) if $\delta^{\downarrow}(g', s) = \mathsf{Unknown}$, then $s \notin X$. Assume that $s \in X$. Then, by P6, there is a weakly feasible mapping $F' \colon V(G|g') \to V(\hat{N})$ for (g', s, X). Since, $F'^{*}(g) \preceq s$, there must be $v \in V(\hat{N})$ such that $F'(g) \preceq v$ and $\pi(v) = s$. Now, let $F := F'|_{V(G|g') \setminus \mathsf{UDup}(F')} \cup \mathsf{UDup}(F') \times \{v\}$. It is not difficult to see that F is feasible for (g', s, X), since all duplications from $\mathsf{UDup}(F')$ are assigned to s. A contradiction.

(A2) If $\epsilon(g', s) = \mathsf{Unknown}$, then there is a weakly feasible F' for (g', s, X) and g' is an lca-duplication assigned to s. Here, $\sigma(g', s)$ cannot be True, thus $\sigma(g', s) = \mathsf{False}$, by P3 and P4. Therefore, $\delta(g', s) = \mathsf{Unknown}$. The rest follows from P2.

We first prove properties P1-P4 for δ and σ. Then, we show that P5 and P6 hold for g and s.

(P1, $\Rightarrow$): If $\delta(g, s) = \mathsf{True}$, then, from (3) g is internal, $s \in X$ and $\delta^{*}(g, s) = \mathsf{True}$. Then, w.l.o.g., for a child g' of g, $\epsilon(g', s) \wedge \delta^{\downarrow}(g'', s) = \mathsf{True}$. Since, $\delta(g', s) \vee \sigma(g', s)$ is True, it follows from the inductive assumption for P1 and P3, that there is a feasible mapping F' for (g', s, X) such that $F'^{*}(g') = s$ and g' is either speciation or lca-duplication. For the other child, we have $\delta^{\downarrow}(g'', s) = \mathsf{True}$. From P5, there is a feasible mapping F'' for (g'', s, X). Then, the mapping $F' \oplus F''$ is feasible for (g, s, X) where g is an lca-duplication assigned to s.

(P1, $\Leftarrow$): Assume there is a feasible mapping $F \colon V(G|g) \to V(\hat{N})$ for (g, s, X) such that g is an lca-duplication assigned to s. Thus, $s \in X$ and g is internal. We conclude that the condition from (3) is satisfied, and $\delta(g, s) = \delta^{*}(g, s)$. W.l.o.g. we assume that g' is assigned to s (recall that g is an lca-duplication). Then, $F|g'$ is feasible for (g', s, X). By the inductive assumption for P1 (if g' is a duplication) or P3 (if g' is a speciation or a leaf), we conclude that $\epsilon(g', s) = \mathsf{True}$. For the second child, we have $F|g''^{*}(g'') \preceq s$, thus $F|g''$ is feasible for (g'', s, X) and by P5, $\delta^{\downarrow}(g'', s) = \mathsf{True}$. Finally, $\delta^{*}(g, s) = \mathsf{True} = \delta(g, s)$.

(P2, $\Rightarrow$): Let $\delta(g, s) = \mathsf{Unknown}$.

(Case P2.a) If $s \in X$ then, from (3) g is internal and $\delta^{*}(g, s) = \mathsf{Unknown}$. W.l.o.g., we may assume that $\epsilon(g', s) \wedge \delta^{\downarrow}(g'', s) = \mathsf{Unknown}$, thus $\delta^{\downarrow}(g'', s) \succeq \mathsf{Unknown}$. If $\delta^{\downarrow}(g'', s) = \mathsf{Unknown}$ then $s \notin X$ from (A1), we conclude that $\delta^{\downarrow}(g'', s) = \mathsf{True}$ and $\epsilon(g', s) = \mathsf{Unknown}$. By (A2) there is a weakly feasible mapping F' for (g', s, X) and g' is an lca-duplication assigned to s. Since $s \in X$, we can construct a feasible mapping for (g', s, X) from a weakly feasible F', by assigning all upper duplications from F' to s. A contradiction.

(Case P2.b) Assume that $s \notin X$. Then, from (4) g is internal and $\delta^{*}(g, s) \wedge \mathsf{Unknown} = \mathsf{Unknown}$. We have that $\delta^{*}(g, s) \in \{\mathsf{True}, \mathsf{Unknown}\}$. (Case P2.b.1) Let $\delta^{*}(g, s) = \mathsf{True}$. W.l.o.g. assume that $\epsilon(g', s) = \delta^{\downarrow}(g'', s) = \mathsf{True}$. Then, $\epsilon(g', s) = \sigma(g', s) \vee \delta(g', s) = \mathsf{True}$. From P1 and $s \notin X$, $\delta(g', s)$ cannot be True, thus $\sigma(g', s) = \mathsf{True}$. From P3, there is a feasible F' for (g', s, X) and g' is an speciation or a leaf assigned to s. For the second child we have $\delta^{\downarrow}(g'', s) = \mathsf{True}$ and from P5, there is a feasible F'' for (g'', s, X). Let $F = F' \oplus F''$. It is not difficult to see that g is an lca-duplication in F since g' is speciation or a leaf. Thus, F is weakly feasible. Note that there is no feasible mapping for (g, s, X); otherwise, $x \in X$ based on the property that g is an lca-duplication assigned to s.

(Case P2.b.2) Let $\delta^{*}(g, s) = \mathsf{Unknown}$. W.l.o.g. assume that $\epsilon(g', s) \wedge \delta^{\downarrow}(g'', s) = \mathsf{Unknown}$. If $\epsilon(g', s) = \mathsf{True}$ then $\delta^{\downarrow}(g'', s) = \mathsf{Unknown}$. Then, similarly to the previous case g' is a speciation or a leaf assigned to s, and there is a feasible F' for (g', s, X), while, from P6, there is a

weakly feasible F'' for (g'', s, X). Then, similarly to the previous case $F' \oplus F''$ is weakly feasible mapping for (g, s, X) where g is an lca-duplication assigned to s. It remains to analyse the case when $\epsilon(g', s) = \mathsf{Unknown}$. By (A2) there is a weakly feasible F' for (g', s, X) and g' is an lca-duplication assigned to s. Here, $\delta^{\downarrow}(g'', s) \in \{\mathsf{True}, \mathsf{Unknown}\}$, and depending on the value either, by P5 there is a feasible (True) or, by P6, weakly feasible ($\mathsf{Unknown}$) F'' for (g'', s, X). In the valid mapping $F' \oplus F''$, g' is an lca-duplication assigned to s, and g is also lca-duplication assigned to s. However, $s \notin X$, thus the mapping is weakly feasible for (g, s, X). Also, there is no feasible mapping for (g, s, X) in this case, due to the property that g' is an lca-duplication assigned to s.

(P2, $\Leftarrow$). Let F be a weakly feasible mapping for (g, s, X) such that g is an lca-duplication assigned to s. Since g is an lca-duplication, g is also an upper duplication and $s \notin X$. Thus, $\delta(g, s) = \delta^*(g, s) \wedge \mathsf{Unknown}$ from (4). W.l.o.g., we may assume that g' is assigned to s. If g' is a speciation or a leaf, then $F|g'$ is feasible for (g', s, X) since no upper duplication is present in $G|g'$. From P3, $\sigma(g', s) = \epsilon(g', s) = \mathsf{True}$. If g' is a duplication, g' is an upper lca-duplication, thus $F|g'$ is weakly feasible. From P2, $\delta(g', s) = \mathsf{Unknown}$. In all cases, $\epsilon(g', s) \geq \mathsf{Unknown}$. Similarly, for the second child of g, the mapping $F|g''$ is either weakly feasible for (g'', s, X) if g'' is an upper duplication assigned to a node not in X, or feasible otherwise. By P5 and P6, $\delta^{\downarrow}(g'', s) \geq \mathsf{Unknown}$. Finally, $\delta^*(g, s) \geq \mathsf{Unknown}$ and $\delta(g, s) = \delta^*(g, s) \wedge \mathsf{Unknown} = \mathsf{Unknown}$.

(P3, $\Rightarrow$). Let $\sigma(g, s) = \mathsf{True}$. Note that at least one of g and s is internal by the inductive assumption. Then, g is internal and s is a tree-node, from (9). W.l.o.g. we may assume that $\delta^{\downarrow}(g', s') \wedge \delta^{\downarrow}(g'', s'') = \mathsf{True}$. Thus, from P5, we have two feasible mappings F' for (g', s', X) and F'' for (g'', s'', X). Then, the mapping $F' \otimes F''$ is feasible for (g, s, X) where g is a speciation assigned to s.

(P3, $\Leftarrow$). Assume that there is a feasible mapping F for (g, s, X) such that g is a speciation or a leaf assigned to s. If g is a leaf, the statement is obvious. Assume that g is a speciation, then s is a tree-node and $\sigma(g, s)$ follows from (9). W.l.o.g. we may assume that $F^*(g') \preceq s'$ and $F^*(g'') \preceq s''$. Thus, $F|g'$ is feasible for (g', s', X). From P5, $\delta^{\downarrow}(g', s') = \mathsf{True}$. Similarly, we obtain $\delta^{\downarrow}(g'', s'') = \mathsf{True}$. Finally, $\sigma(g, s) = \mathsf{L}(\delta^{\downarrow}(g', s') \wedge \delta^{\downarrow}(g'', s'')) = \mathsf{True}$.

(P4) It follows easily from the definition of σ and the operator L.

(P5, $\Rightarrow$) Assume that $\delta^{\downarrow}(g, s) = \mathsf{True}$.

(Case P5.1) If s is a leaf, then g is internal by the inductive assumption. Then, by (8), $\delta^{\downarrow}(g, s) = \epsilon(g, s) = \delta(g, s) \vee \sigma(g, s) = \mathsf{True}$. Note, that $\sigma(g, s) = \mathsf{False}$, otherwise both g and s are leaves. Thus, $\delta(g, s) = \mathsf{True}$ and the feasible mapping for (g, s, X) exists by the already proven P1.

(Case P5.2). If s is a tree-node and $s \notin X$, then, by (8), one of $\delta(g, s)$, $\sigma(g, s)$, $\delta^{\downarrow}(g, s')$ or $\delta^{\downarrow}(g, s'')$ is True. If $\delta(g, s) = \mathsf{True}$, there is a feasible mapping for (g, s, X) from already proven P1 for g and s. Similarly, we have the mapping from P3 if $\sigma(g, s) = \mathsf{True}$. If $\delta^{\downarrow}(g, s') = \mathsf{True}$, then there is a feasible mapping F for (g, s', X) from the inductive assumption for P5. Clearly, F is also feasible for (g, s, X). The remaining case when $\delta^{\downarrow}(g, s'') = \mathsf{True}$ is analogous.

(Case P5.3). If s is a tree-node and $s \in X$, then, by (7), at least one among $\delta(g, s)$, $\sigma(g, s)$, $\mathsf{M}\,\delta^{\downarrow}(g, s')$, and $\mathsf{M}\,\delta^{\downarrow}(g, s'')$ is True. The proof is the same as above when $\delta(g, s) = \mathsf{True}$, $\sigma(g, s) = \mathsf{True}$, $\delta^{\downarrow}(g, s') = \mathsf{True}$ or $\delta^{\downarrow}(g, s'') = \mathsf{True}$. For the remaining case, assume that $\delta^{\downarrow}(g, s') = \mathsf{Unknown}$. Then there is a weakly feasible mapping F for (g, s', X) from the inductive assumption for P6. By reassigning all upper F-duplications to s we construct a feasible mapping for (g, s, X). The remaining case when $\delta^{\downarrow}(g, s'') = \mathsf{Unknown}$ is analogous.

(P5, $\Leftarrow$) Assume there is a feasible mapping F for (g, s, X).

(Case P5.1) If s is a leaf, then g is internal by the inductive assumption. Thus, g is an upper lca-duplication assigned to s under the mapping. Thus, $s \in X$ and by already proven P1, $\delta(g, s) = \mathsf{True}$. This yields $\epsilon(g, s) = \mathsf{True} = \delta^{\downarrow}(g, s)$ in this case by (8).

(Case P5.2) Let s be a tree-node. If g is an lca-duplication assigned to s then similarly to the above case, from P1, $s \in X$, and $\delta(g, s) = \mathsf{True}$ and $\delta^{\downarrow}(g, s) = \mathsf{True}$ using (7). The proof is

analogous when g is a speciation assigned to s. The only difference is that s need not be in X.

For the remaining cases, we have either g is a duplication assigned to s, but not the lca-duplication, or there exists $v \prec s$, such that g is either a duplication, or a speciation assigned to v.

(Case P5.2.a) Let g be a speciation assigned to a node v in N. W.l.o.g., we may assume that $v \preceq s' \prec s$. Then $F^*(g) = v$ and there is no upper duplication in T. Clearly, F is also feasible for (g, s', X). From, the inductive assumption for P5, $\delta^{\downarrow}(g, s') = \mathsf{True}$ and also $\mathsf{M}\,\delta^{\downarrow}(g, s') = \mathsf{True}$. In all cases, from (7) if $s \in X$, and (8), otherwise, we obtain $\delta^{\downarrow}(g, s) = \mathsf{True}$.

(Case P5.2.b) Assume that g is a duplication assigned to s, but not the lca-duplication. Thus, $s \in X$ and $F^*(g) = s$. Let $w := \mathsf{M}_{\xi}(g) \in \hat{N}$, where $\xi = F|_{L(G)}$. In other words, w is the lca-mapping of g in $\hat{N}$. Since, g is not lca-duplication, we have that $\pi(w) \prec s$. Let v' be a child of $F(g)$ present on the path from $F(g)$ to v in $\hat{N}$. Then, $\pi(v')$ is a child, say s', of s in N. We construct a mapping F' such that $F'(u) := v'$, if u is upper duplication induced by F, and $F'(u) := F(u)$, otherwise. If $s' \in X$, then such a mapping is feasible for (g, s', X) since all upper duplications are assigned to s', otherwise, the mapping is weakly feasible for (g, s', X). Note that in the second case, there may exist alternative feasible mappings for (g, s, X'). In both cases, we get from the inductive assumption for $P5$ and $P6$ that $\delta^{\downarrow}(g, s') \geq \mathsf{Unknown}$, which gives $\mathsf{M}\,\delta^{\downarrow}(g, s') = \mathsf{True}$ (note that $s \in X$). Thus, $\delta(g, s) = \mathsf{True}$.

(Case P5.2.b) Assume that g is a duplication assigned to a node strictly below s. Similarly to the previous case, we define w and identify s' as the child of s such that there is a path from s' to $\pi(w)$. We conclude that $F^*(g) \preceq s'$. Thus, F is a feasible mapping for (g, s', X). The rest follows in the same way.

(P6, $\Rightarrow$) Assume, $\delta^{\downarrow}(g, s)$ is $\mathsf{Unknown}$. Here, $\mathsf{Unknown}$ is obtained only from (8) (i.e. when $s \notin X$). In addition, s is a tree-node (i.e. $ch(s)$ is not empty). After expanding ϵ, $\delta(g, s) \vee \sigma(g, s) \vee \delta^{\downarrow}(g, s') \vee \delta^{\downarrow}(g, s'')$ is $\mathsf{Unknown}$. By P4, $\sigma(g, s) = \mathsf{False}$. If $\delta(g, s) = \mathsf{Unknown}$, then there is no feasible mapping for (g, s, X) but there is a weakly feasible mapping from already proven P2. If $\delta^{\downarrow}(g, s') = \mathsf{Unknown}$, then, by the inductive assumption for P6, there is no feasible mapping for (g, s', X), but there is a weakly feasible mapping for (g, s', X). Since, $s \notin X$, these properties are also satisfied for (g, s, X). A similar argument hold when $\delta^{\downarrow}(g, s'') = \mathsf{Unknown}$.

(P6, $\Leftarrow$) Assume there is no feasible mapping for (g, s, X), but there is a weakly feasible mapping F for (g, s, X). We show that $\delta^{\downarrow}(g, s) = \mathsf{Unknown}$.

(Case P6.1) If s is a leaf, then g is internal by the inductive assumption. Thus, g is an upper duplication assigned to s. Thus, $s \notin X$ and by already proven P2, $\delta(g, s) = \mathsf{Unknown}$. Since, $\sigma(g, s) = \mathsf{False}$, $\epsilon(g, s) = \mathsf{Unknown}$ and $\delta^{\downarrow}(g, s) = \mathsf{Unknown}$ from (8).

(Case P6.2) Assume s is internal. Note that g cannot be a speciation or a leaf, otherwise there is no upper duplication in T and F is not weakly feasible. If g is an lca-duplication assigned to s, then similarly to the previous case, from P2, $s \notin X$, and $\delta(g, s) = \mathsf{Unknown}$. Note that $\delta^{\downarrow}(g, s') = \delta^{\downarrow}(g, s'') = \mathsf{False}$ (since g is lca-duplication). We conclude that $\delta^{\downarrow}(g, s) = \mathsf{Unknown}$ from (8).

For the remaining case, g is a duplication, and either g is assigned to s and g it is not lca-duplication or g is assigned to a node strictly below s. Also $s \notin X$, otherwise there is a feasible mapping for (g, s, X) obtained from F by assigning all upper duplications to s. The mapping F is weakly feasible for (g, s', X), where s' is the node in N from which $\pi(F(g))$ is visible (see P5.2, $\Leftarrow$ for the construction of s'). Clearly, there is no feasible mapping for (g, s', X), otherwise such a mapping would be feasible for (g, s, X). By the inductive assumption for P6, we conclude that $\delta^{\downarrow}(g, s') = \mathsf{Unknown}$. For the second child of s, we also have $\delta^{\downarrow}(g, s'') \neq \mathsf{True}$, otherwise, by P5, F is feasible for (g, s'', X) and also for (g, s, X), a contradiction. Similarly, we have that $\delta(g, s) \neq \mathsf{True}$ and $\sigma(g, s) = \mathsf{False}$. Thus, $\epsilon(g, s) \leq \mathsf{Unknown}$. Finally, $\delta^{\downarrow}(g, s) = \mathsf{Unknown}$, which follows from (8). This completes the proof of all properties P1-P6 and Lemma 1.

Appendix: the proof of Theorem 1 (from the main article)

Proof. The proof follows immediately from P5 of Lemma 1: $\delta^{\downarrow}(\mathsf{root}(G), \mathsf{root}(N))$ is True if and only if there is a feasible mapping F for $(\mathsf{root}(G), \mathsf{root}(N), X)$. In such a case $\mathsf{Epi}(F) \subseteq X$. □

Appendix: the proof of Lemma 2 (from the main article)

Proof. If there is a feasible solution in the outgrouped case, then there is an episode at the root of network N^{ω}. Removing the inserted nodes yields a feasible solution to the original problem instance. The other direction follows similarly. □

Appendix: Outgroup construction for multiple gene trees (example)

We illustrate the outgroup construction from Lemma 2 (from the main article) on a small instance with two gene trees $G_1 = (a, (b, c))$ and $G_2 = ((a, b), c)$ over taxa $\{a, b, c\}$ and a network N.

Introduce an outgroup species $\omega \notin \{a, b, c\}$. Extend the network to N^{ω} by adding a new root r^{ω} with children ω (leaf) and the original root of N. For each gene tree, append ω as a sibling of its root: $G_1^{\omega} = (G_1, \omega)$ and $G_2^{\omega} = (G_2, \omega)$. Then merge iteratively: $G_2^{\omega} = (G_2^{\omega}, G_1^{\omega})$, so the root of the merged tree has G_2^{ω} and G_1^{ω} as children. This root is mapped to r^{ω} as a speciation, and the two subtrees independently resolve G_1 and G_2 inside N.

Applying Alg. 1 to $(G_2^{\omega}, N^{\omega})$ and removing the forced episode at r^{ω} yields the optimal episodes for the original instance (G_1, G_2, N), as stated in Lemma 2.

Appendix: Lemma 4 (Reticulation Exclusion)

This lemma justifies a key optimization in the DP: since a reticulation node has a unique child in $\hat{N}$, a duplication mapped to the reticulation is indistinguishable from one mapped to its child, so we may always assume episode locations lie outside $R(N)$.

Lemma 4. *For a valid mapping F between G and $\hat{N}$, let F' be the mapping such that for $g \in V(G)$, $F'(g)$ is the child of $F(g)$ if $\pi(F(g)) \in R(N)$, and $F'(g) := F(g)$, otherwise. Then, F' is valid, $\mathsf{Epi}(F') \cap R(N) = \emptyset$ and $|\mathsf{Epi}(F)| \geq |\mathsf{Epi}(F')|$.*

Proof. We show that F' is valid, which follows from the fact that there is no speciation assigned to a reticulation node, since it has a single child. Thus, any node g assigned to a reticulation s via F must be a duplication. Moreover, every child g' of g is either a duplication assigned to s or below, or it is a speciation assigned to the only child s' of s or below. Thus, mappings of all duplications assigned to s can be lowered to be assigned to s'. Now, all four conditions of mapping validity for F' follow easily. □

Acknowledgements

Financial support was provided by the National Science Centre grant #2023/51/B/ST6/02792.
